# Supplementary material for: Are carbon emissions trading and green financial instruments synergistic? -Comprehensive quantitative research based on content analysis
Source: PLoS One. 2024 Mar 7;19(3):e0298601. doi: 10.1371/journal.pone.0298601 (PMC10919601; doi:10.1371/journal.pone.0298601)
Supplement: S1 Data — (ZIP) [file pone.0298601.s001.zip › Data/地级市Z_F工作报告.zip!/╕ú╜¿/─■╡┬╩╨/└┤╫╘╣1⁄2╓┌║┼í╛┬φ┐╦╩2╛▌═°í┐_2016.docx]

各位代表：

　　现在，我代表宁德市人民政府向大会作政府工作报告，请各位代表连同《宁德市国民经济和社会发展第十三个五年规划纲要（草案）》一并审议，并请市政协各位委员和其他列席的同志提出意见。

　　一、2015年和“十二五”时期经济社会发展回顾

　　2015年，在省委、省政府和市委的坚强领导下，我市各级政府深入贯彻落实党的十八大、十八届三中、四中、五中全会和习近平总书记系列重要讲话精神，坚持稳中求进工作总基调，积极应对经济下行压力，深入开展“三比一看”和“三转一加强”活动，强化政策引导，突出项目带动，攻坚克难，铁心拼搏，深入实施“环三”规划，加快推进“六新大宁德”建设，经济社会呈现稳中向好的发展势头。初步统计，全市生产总值1500亿元、增长9.0%；公共财政总收入147.4亿元、增长5.0%，地方公共财政收入104.39亿元、增长5.5%；全社会固定资产投资1288.32亿元、增长11.3%；出口总值242.49亿元、增长7.3%；实际利用外资2.1亿美元、增长20.3%；社会消费品零售总额465.45亿元、增长12.2%；居民消费价格总水平上涨1.5%；城镇居民人均可支配收入25990元、增长8.5%；农民人均可支配收入12432元、增长10%。

　　一年来的主要工作和成效是：

　　（一）着力调优结构，三次产业协调发展。农业基础进一步夯实。以农业农村“五位一体”为抓手，加快提升特色现代农业。实现农林牧渔业总产值444.85亿元，增长4.6%，粮食总产量64.06万吨。新增设施农业1.4万亩、市级以上农业产业化龙头企业131家。茶叶、水产、食用菌、果蔬、林竹、畜禽、中药材等特色产业加快发展。坦洋工夫荣膺米兰世博会中国馆全球合作伙伴。海鸥水产获批建设全国第一家水产类企业国家重点实验室。正冠渔业远洋捕捞船队赴印度洋作业，实现我市远洋捕捞业零的突破。工业大盘总体稳定。组织实施转型升级行动计划和《中国制造2025》行动计划，及时出台促进工业创新转型发展等政策措施，积极帮助企业融资金、拓市场、增效益，实现规模以上工业增加值717.85亿元、增长9.7%。新增产值亿元以上企业19家、产值50亿元以上企业3家、产值100亿元以上企业1家。冶金新材料、新能源、合成革产业逆势增长，对规模以上工业增长贡献率达97%。中国不锈钢行业大会、第六届中国生态合成革论坛在我市成功召开，扩大了行业影响力和集聚力。广生堂药业成功上市，实现了我市民营企业上市A股零的突破。新能源科技、亚南电机获设国家级博士后科研工作站。新增高新技术企业9家、创新型企业9家、发明专利授权166件、驰名商标4件。第三产业加快培育。实现增加值465亿元，增长9.1%。电子商务发展迅速，福安、古田、屏南入选省级农村电子商务示范县，屏南成为阿里巴巴在我市营运的首个农村淘宝县。旅游业进一步升温，接待游客1840.68万人次，增长15%；旅游总收入151.97亿元，增长19.2%。评选产生10项“十佳”旅游品牌，新增周宁陈峭、福安廉村、穆云畲族生态旅游区3个3A级景区。第五届宁德世界地质公园文化旅游节成功举办。金融服务业稳健发展，金融机构本外币存贷款余额1218.85亿元、1400.96亿元，分别增长12.2%和5.6%。房地产市场总体平稳。

　　（二）强化投资引领，项目带动成效显著。组织实施市重点项目380个，完成投资698.7亿元，其中年投资额超10亿元项目10个。列入省行动计划重大项目完成投资221.4亿元，七个领域扩大有效投资完成295.7亿元。宏旺、甬金一期、新能源二期等94个重点项目竣工投产，中聚天冠、三都澳大黄鱼产业园、周宁抽水蓄能电站等103个重点项目开工建设，霞浦核电基地、上白石水利枢纽等一批重大项目前期工作扎实推进。衢宁铁路宁德段开工建设，合福铁路宁德段建成通车；福寿高速、京台高速宁德段、沈海复线柘荣至福安段、宁连高速飞鸾互通至罗源界建成通车，新增高速公路通车里程156.6公里；改造新建普通干线公路236公里，近90%的乡镇实现“镇镇有干线”。电网建设更趋完善，新扩建110千伏及以上变电站12座。

　　（三）突出宜居宜业，城乡建设加快推进。中心城区市政配套不断完善，实施城建项目77个，完成投资22.04亿元。塔南片区、东侨工业集中区路网进一步完善，金马北路、金漳路等市政道路动工建设。新建一批供水、燃气、污水处理、电力管网及公交场站，新增电动公交车85辆。城镇化建设取得实效，福安、屏南省级新型城镇化试点工作启动，29个小城镇综合改革建设试点持续推进，赛岐、太姥山小城市培育试点全面展开，建成一批市政道路、公园绿地、污水垃圾处理设施，镇容镇貌进一步提升。宜居环境建设扎实开展，创建美丽乡村134个，其中示范村13个，打造美丽乡村景观带4条。改造棚户区49万平方米。拆除“两违”建筑物446.34万平方米。完成5个小型农田水利重点县年度建设任务。城乡防灾减灾设施得到加强，除险加固水库62座、海堤15条，治理中小河流12条。

　　（四）深化改革开放，发展活力有效激发。申请创建国家军民融合深度发展试验区工作得到中央领导、军方、国家部委肯定和支持。宁德港区口岸扩大开放继续推进，漳湾作业区水域获准临时开放。深化医药卫生体制改革试点工作有序开展，综合改革覆盖全市公立医院。市医院、闽东医院医疗集团进入实质性运转，蕉城、福鼎分级诊疗试点工作启动。行政审批制度改革持续深化，“三张清单”公布运行，近两年市级共下放委托审批事项148项，基本实现简政放权的预定目标。财税体制改革进程加快，预决算公开范围扩大，政府债务管理进一步规范，政府购买服务有序推进，一批项目运用PPP模式建设。市属国有企业改革深入推进，市国投公司、交投集团、城建集团完成整合重组。福鼎在全省率先实行不动产统一登记试点，市县两级全面设立不动产登记机构。完成新一轮机关事业单位工作人员工资标准调整，城乡居民基本医疗保险政策实现一体化。“三证合一”“一照一码”登记制度全面推行，新增市场主体3.42万户，增长37%。对外交流合作不断拓展，对接签约“三维”项目166个，总投资1278.34亿元。新批外商直接投资和增资项目21个。水产品、茶叶出口预计分别增长16.7%、34%。第六届海峡两岸电机电器博览会成功举办，第七届海峡论坛?陈靖姑文化节被列为国台办重点交流项目。区域协作深入推进，友城联系更加密切。

　　（五）坚持绿色发展，环境质量持续改善。实施重点节能技改项目21个、减排项目156个，年度节能减排任务顺利完成。大气污染防治进一步推进，大气环境质量达到国家二级标准。福鼎热电联产集中供热项目、危废处置中心启动建设，鼎信、联德等企业加大投入，环保整治取得积极成效。淘汰黄标车1821辆。“水十条”加快实施，落实重点流域综合整治项目56个，县级以上集中式饮用水源地水质达标率、主要流域水环境功能达标率均达100%。环保基础设施加快建设，霞浦污水处理厂二期、福安柳堤污水处理厂建成投运，27个重点乡镇实现污水集中处理。造林绿化19.52万亩，治理水土流失19.65万亩。深入开展矿山“一退三清”工作。成立环三绿色发展基金会。柘荣通过国家级生态县验收，寿宁、蕉城、古田通过省级生态县（区）验收。

　　（六）优化公共服务，社会事业加快进步。教育事业优先发展，中小学校扩容工程顺利推进，新增学位5767个。义务教育完小校以上学校标准化建设全面完成，古田、霞浦、寿宁、柘荣通过“义务教育发展基本均衡县”省级评估验收。医疗服务不断提升，医疗机构新增床位408张，市妇幼保健院（含儿童医院）挂牌成立，“海云工程”完成四期推广，在全省率先实现村医养老保险全覆盖。完成省下达的计划生育目标管理责任指标。文化建设成效明显，全国首部畲族题材舞剧《山哈魂》荣获第六届福建艺术节一等奖，畲族银雕工艺研发中心入选国家级特色文化产业重点项目。在全省率先实现农村有线广播应急预警系统四级联播联控。古田、福鼎荣膺全国文化先进县。我市及蕉城、福鼎、古田、霞浦获评省文明城市（县城、城区）。首届全国青运会击剑赛事在我市成功举办，十六届省运会筹备工作扎实推进，市体育中心体育馆等项目启动建设。霞浦被确定为全国青少年校园足球试点县。工、青、妇工作扎实开展，统计、老区、人事编制、民族宗教、档案、老龄、地方志、气象、防震、外事、侨务、残联、移民等工作取得新成效。

　　（七）致力安定稳定，民生福祉不断增进。习近平总书记对福鼎赤溪村扶贫开发工作作出重要批示。全国东部地区扶贫工作座谈会在我市召开，扶贫开发“宁德模式”得到国家层面肯定。6个省级扶贫开发重点县实施扶贫开发项目200多个， 83个省、市扶贫开发重点村实施帮扶项目500多个，完成造福工程搬迁6420户，实现脱贫约3万人。屏南、寿宁列为全国扶贫小额信贷示范县。防抗“苏迪罗”“杜鹃”台风及灾后恢复生产和安置重建工作扎实有效。村（居）委会换届选举工作顺利完成。25项为民办实事项目基本完成，全市民生支出185.19亿元、增长31.2%，占公共财政支出74.8%，同比提高4.2个百分点。新增城镇就业3.39万人、农村劳动力转移就业4.24万人，城镇登记失业率2.62%。1510名高校毕业生实现自主创业。最低工资标准、企业退休人员基本养老金、企业退休军转干部生活困难补贴、城乡居民基础养老金都有新的提高。最低生活保障制度惠及13.56万人。市社会福利中心和5个县级社会福利中心建成。基本建成保障性住房6765套，配租配售4162套。“六五”普法通过验收。各类刑事案件和群体性事件起数明显减少，群众安全感达94.74%。信访总量明显下降。安全生产形势稳定趋好。食品药品安全监管机制逐步完善。“两乱”整治成效显著，宗教领域总体和谐稳定。国防动员、民兵预备役建设、双拥共建、海防、人防和打私工作进一步提升。

　　（八）践行“三严三实”，作风建设得到加强。“三严三实”专题教育深入开展。严格落实中央八项规定精神，“三公”经费下降19.3%。依法行政全面推进，成立政府法律顾问团，建立健全府院、府检互动机制。推动行政执法体制改革，建立行政执法与刑事司法衔接机制，扎实开展相对集中行政处罚权工作。自觉接受人大及其常委会监督并报告工作，依法执行人大及其常委会决议、决定，积极配合开展执法检查、代表视察、专项调研、工作评议和专题询问，办理市人大代表建议278件。支持政协履行政治协商、民主监督、参政议政职能，定期向政协通报情况，积极采纳委员调研成果和协商建议，办理市政协提案207件。主动接受司法、社会和舆论监督。市、县政府机构改革全面完成。机关效能建设和绩效管理得到加强。行政监察和审计监督力度加大，廉政建设和反腐败工作扎实推进。

　　2015年各项目标任务的基本完成，标志着宁德“十二五”规划顺利收官。“十二五”时期，是宁德发展迎来重大历史机遇的五年，是撤地建市以来进步最快、发展最好的五年。这五年，是宁德综合实力迈上新台阶的五年。主要经济指标提速晋位，全市生产总值突破千亿大关，比2010年接近翻一番；公共财政总收入和地方公共财政收入先后突破百亿大关，均比2010年翻一番以上，实现在全省位次前移；规模以上工业总产值突破3000亿大关，是2010年的3.3倍。实现了规划提出的“与全省平均水平差距明显缩小”“在海西发展格局中位次前移”的“两个力争”目标。这五年，是宁德产业发展取得新成效的五年。三次产业结构进一步优化。传统农业加速向特色现代农业转变，新型农业经营主体不断壮大，休闲农业与乡村旅游等新业态蓬勃发展。工业化进程加快推进，规模以上和产值亿元以上工业企业数分别是2010年的1.3倍和2.2倍，培育形成6个百亿产业，特别是冶金新材料和新能源产业从无到有、从小到大、快速发展，千亿产业集群初具规模。现代物流、商贸、金融、电商等服务业加快发展，特别是旅游业发展迅速，接待游客、旅游收入均比2010年翻一番以上，宁德对外知名度进一步提升。这五年，是宁德基础设施实现新突破的五年。固定资产投资累计完成4481.39亿元，是“十一五”的3.5倍，一大批能源、交通、市政、农业、通信基础设施加快建设。电力装机总容量净增350万千瓦，占全省新增容量近四分之一，在全省率先跨入“核电时代”；新增高速公路通车里程241公里，实现“县县通高速”，新增铁路通车里程19.6公里、普通公路通车里程1075公里、港口吞吐能力732万吨；新建城市主干道190公里，完成海堤强化加固64.4公里、水库除险加固186个，新增围垦面积6300亩；实现建制村“村村通宽带”。这五年，是宁德城乡面貌得到新提升的五年。中心城区拓展到34平方公里，人均道路面积17.7平方米，人均公园绿化面积16.5平方米，成功创建国家园林城市；市区及县（市）城镇建成一批城市综合体，城市功能品位进一步提升；县域特色经济加快培育，新增省级生态县8个，小城镇、美丽乡村建设亮点纷呈，城乡环境更加优美，生态环境质量继续位居全省前列；全市常住人口城镇化率53.9%、户籍人口城镇化率34.3%，分别比“十一五”末提高6个和3.5个百分点。这五年，是宁德民生事业取得新进步的五年。民生项目累计投入628.26亿元，是“十一五”的3.4倍。城乡居民人均可支配收入分别比2010年增加9175元、5890元。6个省级扶贫开发重点县经济实力大幅提升，452个贫困村面貌得到较大改善，全市贫困低收入人口从2010年的20.3万减少到11.5万。完成造福工程搬迁27695户11.25万人，建设保障性住房4.17万套。140万农村居民饮水安全问题得到解决。“双高普九”提前实现，义务教育“基本均衡”全面推进，“海云工程”覆盖大部分农村人口，城乡公共文化服务体系初步形成，就业和社会保障体系更加健全。社会治理水平逐年提升，综治考评跃居全省前列。

　　“十二五”发展成就来之不易，这是中央和省委、省政府正确领导、关心支持的结果，是在市委坚强领导下全市干部群众团结一心、奋力拼搏的结果，是市人大、市政协加强监督、鼎力支持的结果。在此，我代表市人民政府，向全市人民，向人大代表、政协委员、各民主党派、工商联、各人民团体、无党派人士、离退休老同志和社会各界人士，向中央及省属驻宁机构、驻宁部队、武警官兵、公安民警，表示衷心的感谢和崇高的敬意！

　　在看到成绩的同时，我们也清醒地认识到发展还面临不少困难和问题，主要是：经济总量仍然偏小，实体经济发展困难增多，融资难、融资贵问题突出，金融风险防范形势依然严峻，稳增长任务艰巨；产业结构不够优，创新能力比较弱，传统产业转型步伐偏慢，新兴产业和第三产业培育不足，环境刚性约束趋紧，节能减排压力增大；城乡规划建设水平不高，管理不够精细，部分地区脏乱差和城区交通拥堵等现象仍较突出；脱贫攻坚任务依然繁重，剩下的都是难啃的“硬骨头”，亟需攻坚拔寨；教育、卫生等公共服务水平与群众期望还有一定差距，社会治安、安全生产等领域还存在一些隐患；依法行政水平有待提高，机关作风效能建设还要进一步深化。对此，我们要高度重视，采取更加有力措施加以解决。

　　二、实施“十三五”规划，确保如期全面建成小康社会

　　“十三五”是实现党中央确定的第一个百年奋斗目标、全面建成小康社会的决胜阶段，也是我市科学发展跨越赶超的关键时期。根据党的十八届五中全会、省委九届十五次全会和市委三届十七次全会精神，市政府组织编制了《宁德市国民经济和社会发展第十三个五年规划纲要（草案）》（以下简称规划草案），提请本次大会审议。规划草案提出了今后五年经济社会发展的总体要求、目标任务和重大措施。

　　“十三五”经济社会发展的总体要求是：全面贯彻党的十八大、十八届三中、四中、五中全会和省委九届十五次全会、市委三届十七次全会精神，深入贯彻习近平总书记系列重要讲话精神和对福建对宁德工作的重要指示，坚持“四个全面”战略布局，坚持发展第一要务，以科学发展为主题，以转型升级为主线，以提高经济发展质量效益为中心，着力创新发展、协调发展、绿色发展、开放发展、共享发展，加快形成适应经济新常态的体制机制和发展方式，全面实施“环三”规划，推进“六新大宁德”建设，推动经济社会发展再上一个新台阶，确保如期全面建成小康社会。

　　规划草案提出经济总量、质量效益、社会民生、生态文明等四大类33项指标，其中，地区生产总值年均增长9%以上，高出全省平均水平约0.5个百分点，到2020年达2350亿元，进一步向全省中游水平迈进；人均生产总值约8万元；居民人均可支配收入超3万元。这些目标，紧扣中心任务、突出理念引领、坚持问题导向、注重上下衔接，经过全市人民共同努力是可以实现的。

　　实现预期目标，我们要重点把握好七个方面：

　　（一）打造宁德产业升级版。认真落实产业转型升级行动计划，着力构建高质高效新产业的“大宁德”。坚持做优一产，用工业方式发展特色现代农业，促进农业与二三产业融合发展，持续打造茶乡、菌都、药城、果竹和水产大市。坚持做强二产，着力实施《中国制造2025》行动计划，推动新兴产业扩容崛起、传统产业升级换代，打造八大特色产业基地，培育4个千亿产业集群和10个以上百亿企业。坚持做大三产，把发展服务业作为补短板、促转型的重要抓手，着力发展旅游、健康养老、文化创意等生活性服务业，加快发展物流、金融、电商等生产性服务业。

　　（二）强化基础设施支撑力。突出重点，组织实施“八大投资工程”。围绕构建畅通便捷新交通的“大宁德”，加快发展大港口、大通道、大物流，着力建设铁路、公路、水路、航空等现代综合交通运输体系。推进一批核电、风电、抽水蓄能等重大能源项目，打造海西多元化能源基地。加快实施上白石水利枢纽、主城区生态补水、溪南半岛引调水等一批重大水利工程，不断完善水利和防灾减灾体系。加快“数字宁德”建设，以加速宽带化、网络化进程和智慧化应用为重点，推进云计算中心建设，完善信息化基础设施。

　　（三）加快推进新型城镇化。遵循城市发展规律，按照“五个统筹”要求，强化规划引领，推进城市发展，着力构建宜业宜居新城镇的“大宁德”。提升中心城市首位度，加快建设连通“一城四区”城市主干道，推动中心城市向临海布局、环湾发展。推进海绵城市建设，完善市政基础设施，打造地标建筑、特色街区，提升城市功能品位。坚持以城促产、强产兴城，统筹推进县域新区拓展、旧城改造，完善基础设施服务功能，提升综合承载能力。加快形成一批工业重镇、旅游名镇、商贸大镇、生态强镇和文化古镇，打造一批美丽乡村景观带，构建城乡一体化和区域协调发展格局。加快农业转移人口市民化，力争2020年全市户籍人口、常住人口城镇化率分别达43%和60%。

　　（四）坚决打赢脱贫攻坚战。总结提升“宁德模式”，解决好“扶持谁”“谁来扶”“怎么扶”问题，组织实施“六百千万”工程，落实“五个一批”要求，深入推进精准扶贫、精准脱贫，五年统筹13亿元用于扶持省级扶贫开发重点县，确保2020年省级扶贫开发重点县全部“脱帽”，452个贫困建制村整村脱贫，1022个边远偏僻自然村完成整村搬迁安置，11.5万农村低收入人口稳定脱贫，实现“两不愁、三保障”。

　　（五）激发改革开放新活力。主动融入“一带一路”和福建海丝核心区建设，着力打造海上丝绸之路由东南沿海向内陆延伸拓展的重要衔接点。推进国家军民融合创新示范区建设，完善“一港五区”布局，加快连接内陆省份战略通道、域内互联互通快速通道建设，形成以三都澳国际化大港为核心的集疏运体系。推动军民融合产业园建设，加快“民参军”“军转民”步伐。实施人才优先战略，推动大众创业万众创新。以制度创新为核心，实施创新驱动五大工程。积极对接福建自贸试验区，营造简政放权、放管结合、优化服务的良好环境。创新利用外资方式和工作机制，加快出口行业转型升级，提高特色品牌海外影响力，构建开放合作新格局。

　　（六）构筑生态文明示范区。深入实施生态市战略，严守生态红线，严格主体功能定位，科学划定生产、生活、生态空间管制界限，优化国土空间开发格局。注重源头管控，持续推动大气、水、土壤污染防治，提高环境监管水平。加强重点行业环保节能改造，严把节能减排关，严控高污染、高排放项目。强化生态文明绩效考评，推行领导干部自然资源资产离任审计、重点流域生态补偿机制，实现省级生态文明建设示范县全覆盖，打造优质优美新环境的“大宁德”。

　　（七）推动公共服务均等化。加大财政投入，加强保障和改善民生，不断增进人民福祉，构建幸福和谐新民生的“大宁德”。坚持教育优先发展，促进教育公平，全面实施“两免一补”，大力发展学前教育，推进义务教育优质均衡发展，普及高中阶段教育。加强社会主义核心价值体系建设，深化精神文明创建活动，大力弘扬“闽东之光”，实施“魅力闽东”文化惠民工程，着力构建丰富多彩新文化的“大宁德”。加大公共卫生服务力度，提升全民健康保障水平。办好十六届省运会。实施城乡居民收入倍增计划，实现更高质量就业创业，缩小城乡、区域、行业收入差距。全面建成覆盖城乡居民的社会保障体系，推进健康养老、医疗保险从制度全覆盖向人员全覆盖转变。推进依法治市，创新社会治理，确保人民群众安居乐业。

　　三、扎实做好2016年工作

　　今年是全面建成小康社会决胜阶段的开局之年，也是推进结构性改革的攻坚之年。我们要深化对经济新常态的认识，牢固树立创新、协调、绿色、开放、共享“五大理念”，坚持稳中求进工作总基调，落实去产能、去库存、去杠杆、降成本、补短板“五大任务”，组织实施重点项目推进年、创新创业提升年、改革任务落实年、服务发展深化年“四个年”活动，着力加强供给侧结构性改革，加快培育新的发展动能，增强持续增长动力，为“十三五”发展再上新台阶开好局、起好步。

　　经济社会发展的主要预期目标是：全市生产总值增长9%；公共财政总收入、地方公共财政收入均增长6%；全社会固定资产投资增长18%；外贸出口增长5%，实际利用外资增长10%；社会消费品零售总额增长12%，居民消费价格总水平涨幅控制在3%以内；城乡居民人均可支配收入分别增长9%；城镇登记失业率控制在4%以内；人口自然增长率控制在9‰以内；完成单位生产总值能耗、化学需氧量、二氧化硫、氨氮、氮氧化物等年度节能减排任务。

　　实现上述目标，重点抓好以下八个方面工作：

　　（一）加快产业转型升级

　　把握转型路径。坚持抓龙头、铸链条、建集群，推动新兴产业发展。加快实施鼎信、联德、宏旺、甬金、新能源科技、宁德核电等6个新增产值10亿元以上项目，推动冶金新材料建成千亿产业集群；以先进产能和关键技术为牵引，扎实实施柘荣药业、古田药业等156个新增产值2000万元以上项目，推动新能源、生物医药行业做大做强。坚持抓整合、重创新、促提升，加快传统产业转型升级。电机电器、船舶产业要抢抓机遇，化解金融风险和过剩产能，推动兼并重组，促进与高科技嫁接、与设计联姻、与品牌联动，实现结构优化、质效提升。以亚南电机、安波电机为龙头，加快发展伺服电机、新能源驱动电机，实现产品升级换代；以白马船厂、福宁重工为龙头，加快发展海洋工程装备、军民融合船舶、高技术船舶，支持长兴重工建设华东地区船舶综合保障动员中心。引导合成革、化油器产业实施清洁生产，推进新工艺技术应用和产业链延伸。

　　增强转型动力。注重供给侧结构性改革，强化创新对提高供给质量的支撑。大力推进智能制造，组织实施18个国家、省级智能制造重点项目。对接和推广“数控一代”，促进数控技术和智能装备应用，力争“机器换工”100台套以上。支持企业开展工业控制软件、控制系统集成等关键技术的研发和产业化应用。推动产学研用协同创新，推进众创空间和科技企业孵化器建设，积极发展众创、众包、众扶、众筹模式。组织实施榕屏化工、安发生物等100项重点技术改造项目，支持科技成果转化、生产装备升级。加强高层次、高技能人才队伍建设，培养科技创新创业领军人才。加强知识产权创造、运用和保护。

　　优化转型环境。落实好稳增长调结构各项政策措施，深化“三比一看”“三转一加强”活动，“一业一策”“一企一策”帮助企业协调解决具体问题。实施降低实体经济企业成本行动，切实降低企业制度性交易、税费、财务、电价、物流等成本，提高供给效率。引导金融机构加大对实体经济的支持和服务力度，推动三祥新材等龙头企业上市，鼓励企业在“新三板”和海交中心挂牌融资。推进园区标准化建设，加快霞浦高科技机械装备制造园区等13个园区的规划修编，推进湾坞西片区污水处理厂等54个园区基础设施项目和福鼎危废处置中心等17个服务设施项目建设，完善园区功能，促进产业集约集聚发展。

　　（二）切实加强“三农”工作

　　提升特色现代农业。强化粮食安全保障，深化粮食高产创建活动，确保粮食播种面积稳定在199万亩，粮食产量稳定在63万吨以上。加快转变农业发展方式，大力发展精深加工，完善“种养加销”链条，做大做强茶叶、水产、食用菌、果蔬、林竹、畜禽、中药材等特色产业。扶持壮大市级以上农业产业化龙头企业，新增产值亿元以上企业10家。加快现代农业示范园区建设，引导设施农业向园区基地聚集，新增设施农业1万亩以上。加强农产品品牌培育，推进农业标准化生产。加快培育农旅结合、农村电商、农产品定制等农业新业态、新模式。

　　完善农村基础设施。加强农田水利基础设施建设，建设高标准基本农田4.5万亩、复耕改造山垅田1.8万亩。加快推进上白石水利枢纽、官昌水库、管阳溪跨流域引水工程等一批重大水利项目建设。继续开展中小河流治理、农村饮水安全巩固提升工程，完成海堤除险加固12条、小型水库除险加固17座。推进5个小型农田水利重点县建设。加强防汛抗旱防灾减灾工作，抓好重大动植物疫病防控。推进水电农村电气化县、小水电代燃料工程建设和农村水电增效扩容改造。加快实施宽带入乡进村工程，继续推进农村公路提级改造。

　　创新农业经营方式。全面推进农村土地承包经营权确权登记颁证工作，推动土地经营权依法规范有序流转。壮大多元经营主体，支持大中专毕业生、返乡人员领办合作社和家庭农场，新增市级以上农民合作示范社50家、家庭农场示范场20家。深化林业配套改革，提高林分质量，发展林下经济。强化农业科技支撑，实施现代种业创新工程，加强农业“五新”集成应用，推广“五新”项目100个。

　　（三）着力实施脱贫攻坚

　　健全脱贫工作机制。全面推行“六个百分之百”挂钩帮扶制度，落实精准扶贫到户各项政策措施，摸清底数、精准识别、分类指导、精准施策，逐户“建档”、逐村“监测”、逐县“挂图”，力争全年减贫3万人。凝聚全社会扶贫合力，加大扶贫资金整合监管力度，市本级新增财力80%以上用于支持贫困县乡村发展。支持屏南开展全国农村改革试验区扶贫开发综合改革试验工作。层层签订脱贫攻坚责任状，建立贫困户脱贫退出认定机制，强化脱贫攻坚成效考评，评选表彰一批“扶贫团队”“扶贫先锋”“脱贫之星”。

　　增强内生发展动力。坚持因地制宜，走开发式扶贫路子，依托区位条件、资源禀赋、产业基础和种养习惯，培育壮大优势特色产业。实施贫困村“一村一品”产业推进行动，引导龙头企业与贫困村开展结对帮扶，合作开发现代农业项目，让贫困户更多分享农业全产业链和价值链增值收益。大力发展集体经济，增加村财收入，力争培育村财收入10万元以上的村200个。坚持治贫先治愚、扶贫先扶智，加强教育扶贫和技能培训，开展新型农民培训2万名、“雨露计划”培训5000名。

　　加快造福工程搬迁。全年计划对300个边远偏僻自然村实施整村搬迁，力争搬迁群众2万人。加强集中安置区基础设施和公共服务配套建设，确保群众搬得出、留得住、能致富。鼓励引导搬迁村落开展旧村改造和宅基地复垦，盘活搬迁群众原有耕地、茶果园、山林等资源。继续支持苏区、老区基点村、少数民族聚居区、海岛、库区等地区加快发展。

　　强化社会保障兜底。统筹协调农村扶贫和低保标准，对贫困人口中完全或部分丧失劳动能力的，由社会保障兜底，实现应保尽保。健全医疗救助机制，确保因病返贫、因病致贫的贫困户得到及时救助。加大对农村留守儿童、留守妇女、留守老人的关爱服务力度。

　　（四）积极扩大有效需求

　　强化投资关键作用。把投资重点放在调结构、补短板、惠民生上，切实提高投资有效性。全年计划实施市级重点项目360个，其中在建项目280个，年度计划投资528.7亿元，力争开工项目90个、竣工项目80个。全力推进衢宁铁路宁德段建设，加快建设海西网屏南至古田联络线、沈海复线福鼎至柘荣段、福安至漳湾段、沙埕湾跨海高速公路、宁德火车站综合客运枢纽和一批普通国省干线公路，建成宁连高速漳湾至飞鸾互通段，启动宁德至古田高速公路工可编制工作；继续推进三都澳港区城澳作业区8#9#泊位、1#2#泊位工程，动工建设漳湾作业区10#泊位工程。加快推进宁德核电5#6#机组、霞浦核电基地、中聚天冠、周宁抽水蓄能项目，新建和改造一批城乡电网。组织实施新能源三期、中铝铜冶炼、鼎信400系、海利不锈钢等一批工业项目。加强项目储备和动态管理，积极向上争取支持，对接和用好国家专项建设基金。完善项目工作机制，加强要素保障，促进项目建设提速增效。

　　发挥消费基础作用。顺应消费需求升级趋势，加快发展“互联网+”，积极培育消费新热点、新模式，促进现代服务业发展。继续培育现代物流业，加强物流节点、园区和配送中心建设，支持快递业发展。着力发展旅游业，整合提升旅游景区和线路，打造旅游龙头，构筑特色旅游网络，促进旅游全域化。办好第六届宁德世界地质公园文化旅游节。引导合理住房消费，适时出台“促销售、去库存”政策，促进房地产业平稳健康发展。

　　增强出口带动作用。积极融入“一带一路”建设，主动对接福建自贸试验区，深化宁德品牌“一带行”“海丝行”活动，拓展东南亚、俄罗斯等沿线国际市场。推进三都澳港区漳湾作业区和三沙港区口岸开放，抓好白马港区口岸联检中心建设。扶持一批外贸公共服务平台与外贸综合服务企业。推进电机电器、农水产品等重点出口行业转型升级，培育不锈钢、合成革等出口新增长点，促进出口产品结构优化。

　　（五）统筹推进城乡建设

　　提升中心城市形象。注重城市设计，发挥规划引领作用，深化滨海新城规划，推进滨海大道内侧围填海和三屿、飞鸾片区围填海，拓展中心城市空间。组织实施136个城建项目，推进金漳路、天德路、金侨路等主通道建设，打通闽东中路、金马北路等断头路；加快东区污水处理厂、漳湾污水处理厂及配套管网建设，科学布局和改造地下管网，抓好内河和东湖水域治理；新建供水管网10公里，实施第二自来水厂扩容工程，提升二次供水能力；推进海绵城市建设，实施南大塘、古溪、大寨溪等一批防洪排涝项目，完成曲尺塘、后岗环岛等低洼地带整治；巩固国家园林城市创建成果，推进五大公园和环东湖栈道建设，提升金溪景观带。

　　加快城镇化建设。探索和完善市县空间规划体系，总结福鼎试点经验，在各县（市）逐步推行“多规合一”。实施“大城关”战略，统筹推进县域产业、人居和基础设施建设，提升综合承载能力。加大城中村、棚户区、城乡结合部改造力度，推动新老城区、城乡连片互动发展。深化福安、屏南省级新型城镇化和29个小城镇综合改革建设试点，抓好赛岐、太姥山小城市培育工作，以点带面，推动周边乡镇与试点镇连片发展。深化户籍制度改革，落实居住证制度，有序推进农业转移人口市民化。

　　深化宜居环境建设。着眼“一村一品”“一村一韵”“一村一记忆”，新建130个美丽乡村、12个示范村，打造4条美丽乡村景观带。加强农村垃圾整治，提高无害化处理水平。发展城市综合交通，抓好公交站点、公共停车场、停车泊位和充电桩建设，新增新能源公交车40辆。按中央和省里部署，深入推进城市执法体制改革，改进城市管理工作。强化道路交通整治，抓好城乡结合部、城市出入口、背街小巷市容市貌动态管理和“广告污染”专项清理。持续整治“三边三节点”，保持“清违”高压态势，遏制新增“两违”。

　　（六）大力推进改革开放

　　积极创建国家军民融合创新示范区。争取国家尽快批准设立示范区。抓紧编制军民融合专项规划，以项目为支撑，强化“一对一”跟踪，“点对点”对接，在交通网络体系、军民兼容产业、社会事业军地合作等领域生成落地一批项目。加快建设军民融合产业园区和农副产品供应基地，积极培育军民兼容产业，推动新能源、电机电器、船舶修造发展军民两用高附加值产品。深化与福州总院的医疗合作。以融合为契机，在扩大三都澳水域开放方面取得新突破。

　　推动重点领域改革。扎实抓好《重点改革任务实施规划》各项举措落实。进一步推进简政放权，巩固提升行政审批制度改革成果，全面实施清单管理，优化前置审批，做好下放审批事项的承接和后续监管。深化工商登记制度改革，持续抓好“三证合一”“一照一码”登记制。稳妥推进事业单位分类改革。深化国企改革，支持国企做大做强主业，提高自营能力。全面推行不动产统一登记。加快落实“营改增”扩围工作。深化预算管理制度改革，强化预算绩效管理。积极争取新增地方政府债券、置换债券和省级股权投资基金的支持。完善政府性债务风险预警机制，切实防范债务风险。深入推广政府和社会资本合作（PPP）模式，扩大政府购买服务范围。全面实行居民用水用电阶梯价格制度。

　　扩大对外交流合作。深化“三维”对接，持续推进产业链招商，加大面向军民融合、现代服务业的招商力度，全年签约项目总投资1200亿元以上。加强外事侨务工作，密切与海外侨团、旅外乡亲社团联系交往，做好新华侨和华裔新生代工作，促进侨资回归。深化与港澳台交流合作。办好第七届海峡两岸电机电器博览会和第八届海峡论坛?陈靖姑文化节，推进畲族文化、圆瑛、甘国宝文化、马仙文化对台交流。

　　（七）加强生态环境保护

　　筑牢生态安全屏障。建立环保绩效考核制度和生态环境损害责任终身追究制。执行生态保护红线管控政策，强化自然保护区、重要湿地、饮用水源地等重点生态功能区保护，严厉打击非法违法采矿行为，切实抓好饰面石材矿山关闭转产工作。实施造林绿化15万亩，治理水土流失15万亩。推动柘荣获得国家级生态县命名，蕉城、古田、寿宁、福安、福鼎通过省级生态县（市、区）复核验收命名，实现省级生态县（市、区）全覆盖。

　　推进环保重点整治。实行四级网格化环保监管，清理整顿环保违规项目，落实冶金、修造（拆）船、合成革、石板材、畜禽养殖等重点行业污染整治。打好水、大气、土壤污染防治三大攻坚战。深入实施“水十条”，严格落实“河长制”，加快重点工业园区和城镇污水处理设施建设，加强运行监管，推动城市黑臭水体治理，强化重点流域水环境综合整治。落实《大气污染防治法》，开展中心城区空气环境质量预测预报，加快燃煤锅炉、扬尘、黄标车淘汰、垃圾露天焚烧等专项治理。实施农用地、建设用地土壤环境分级分类管理。强化危险废物、重金属、核与辐射等环境监管。

　　注重节能减排工作。严格落实环保监管“一岗双责”，坚决淘汰落后产能，大力发展非化石清洁能源和可再生能源。加快工业园区集中供热项目建设，推广应用先进适用清洁生产技术。实施合成革、能源、冶金等重点行业节能降耗工程，推进力捷迅药业等企业循环经济试点工作，加大减排项目建设力度，全面完成脱硫、脱硝设施升级改造任务。节约集约利用土地，抓好闲置土地处置和永久基本农田划定工作，严守耕地保护红线。严格海域使用审批，有度有序开发海域资源。强化“三条红线”考核，促进水资源优化配置。

　　（八）致力改善和保障民生

　　提升社会保障水平。建立经济发展和扩大就业联动机制，完善公共就业创业服务体系，统筹抓好高校毕业生、就业困难人员、农业转移劳动力、退役军人等重点群体就业，积极扶持高校毕业生自主创业，新增城镇就业2.8万人。实施全民参保计划，实行城乡居民基本医保一体化和设区市统筹，全面推进机关事业单位养老保险，启动实施公务员工伤保险。建立县以下机关公务员职务与职级并行和乡镇工作补贴制度，推进国有企业负责人薪酬制度改革。积极创建数字化“无欠薪项目部”，构建和谐劳动关系。全面实施临时救助制度，积极推进市县两级社会福利中心建设，加快建立养老服务体系，促进妇女儿童、残疾人事业发展，关爱失独家庭。深入实施保障性安居工程，开工7298套、基本建成3880套。

　　加快教育卫生事业发展。继续实施新一轮学前教育行动计划，加强幼儿园布局规划，新建16所公办幼儿园，新增学位4800个，规范民办幼儿园管理。继续实施城区中小学新建、扩容工程，加大农村薄弱校改造力度，新增学位4650个。提高义务教育学校生均公用经费补助标准。推动屏南、周宁创建全国义务教育发展基本均衡县。推进普高达标晋级，启动示范性高中评估试点。支持宁德师院创建智能制造和创新创业虚拟学院，支持宁德职业技术学院和4所中职学校建设省示范性现代职业院校。完成福鼎、古田特殊教育学校标准化建设。重视发展终身教育和老年教育。深化医药卫生体制改革，完善医疗集团管理，全面开展分级诊疗，鼓励和支持社会资本办医。加快闽东医院门急诊综合楼等项目建设。推进古田、福安、福鼎精神病院新（改、扩）建，做好肇事肇祸精神病人收治。实施同心全科医生特岗人才计划。提升“海云工程”服务实效。加强中医药事业、疾病预防控制、卫生应急处置工作。全面实施两孩政策。积极筹办、备战十六届省运会，加快体育场馆和配套设施建设，加大体育人才培养力度，提高竞技体育运动水平。广泛开展全民健身活动。

　　大力推进文化建设。以培育和践行社会主义核心价值观为主线，大力弘扬“滴水穿石”闽东精神，深化文明城市、文明村镇、文明单位创建工作，推动志愿服务、网络文明传播常态化，提高思想道德和社会诚信水平。弘扬特色传统文化，做大“魅力闽东”文化惠民品牌。优化公共文化服务体系，加快发展新型文化业态，促进传统媒体和新兴媒体融合发展，积极传播正能量、占领主阵地。加快电视台高清改造、电视数字化转换、县（市）城区数字影院建设。积极开展省级畲族文化生态保护实验区示范点创建，完成第一次可移动文物普查，继续做好第二轮修志工作。推进坦洋工夫中国红茶文化产业园、宁德工艺博览城、盈盛号国家文化产业示范基地建设。繁荣文艺创作，打造一批体现闽东特色的文艺精品。

　　加强社会治理创新。持续深化“平安宁德”建设,总结推广蕉城“海上枫桥”和寿宁“无访乡村（社区）”经验，探索推进沿海平安海域、山区平安走廊、行业系统平安领域“三条主线”创建。加快立体化社会治安防控体系建设，落实反恐防范措施，加强禁毒和社区戒毒康复工作，严厉打击各类违法犯罪活动。创新信访工作机制，着力化解一批信访积案。健全社会稳定风险评估和突发公共事件预警应急处置机制。积极防范和化解金融风险，规范民间融资行为，坚决打击非法集资。加强特殊人群和流动人口服务管理。深化道路交通、危险化学品、建筑施工、消防、教育等重点行业领域安全生产专项整治，促进安全生产形势持续稳定向好。加大“餐桌污染”治理力度，推进食品药品安全社会共治。实施“七五”普法，推进依法治市。充分发挥工会、共青团、妇联等人民团体的作用。完善基层群众组织自治制度。落实民族政策，依法管理宗教事务。加强国防动员、民兵预备役、双拥共建和海防、人防、打私等工作。

　　四、着眼“三提高一加强”，切实强化政府自身建设

　　各位代表！起跑决定后势，开局关系全局。各级政府要自觉强化政治意识、看齐意识、带头意识，严守政治规矩和政治纪律，在思想上政治上行动上与党中央保持高度一致，坚决贯彻落实中央和省委、省政府、市委的决策部署，积极应对新挑战，以新的发展理念引领新的发展实践，以新的精神状态开拓新的发展境界。

　　解放思想，提高创新力。自觉践行“三严三实”要求，以严实正作风，推动各项工作。敢于担当、主动作为，解放思想、转变观念，以先行抢占先机、以先试闯出新路。加强学习、与时俱进，坚持理论武装和实践历练并举，更好地认识新常态、适应新常态，牢固树立“五大发展理念”，不断开创工作新局面。完善容错纠错机制，关爱和支持勇于改革、善于创新的干部，为改革创新、干事创业创造更好的政治生态，营造更好的环境氛围。

　　依法行政，提高公信力。坚持党的领导，在法治轨道上开展工作，依法履职，遵循程序，做到“法无授权不可为”“法定职责必须为”。健全依法决策机制，深化行政执法体制改革，规范行政执法行为。依法接受人大及其常委会的法律监督和工作监督，主动接受政协民主监督，认真听取各民主党派、工商联和无党派人士意见，提高人大代表建议、意见和政协提案办理质量。

　　强化落实，提高执行力。面对新形势、新挑战，各级政府要及时跟进应对举措，解决问题不拖延、化解矛盾不积压。发扬“钉钉子”精神，对看准的事情、定下的目标、部署的工作，雷厉风行、一抓到底，确保各项工作落到实处、取得实效。牢固树立政府工作“一盘棋”观念，强化部门之间、上下之间的主动协调、密切配合，提高整体工作效能。坚持“四下基层、四解四促”，推行“一线工作法”，具体实干、马上就办，把问题解决在一线。深化绩效管理和机关效能建设，治理“庸懒散奢”，强化行政问责，优化政务服务环境。

　　加强廉政建设。认真落实党风廉政建设责任制，严格执行《中国共产党廉洁自律准则》《中国共产党纪律处分条例》，让廉洁从政成为一种自觉、一种品格，落实到处理政务、推进发展的各个环节。各级政府要强化主体责任意识，坚持既管事又管思想管作风，使广大公务人员心有所畏、言有所戒、行有所止，努力实现干部清正、政府清廉、政治清明。重视加强重点领域监管和行政监察、审计监督，消除权力寻租空间，确保权力在阳光下运行。

　　各位代表！信心凝聚力量，实干铸就辉煌。让我们紧密团结在以习近平同志为总书记的党中央周围，在省委、省政府和市委的坚强领导下，紧紧依靠全市人民，振奋精神，同心协力，为推动“六新大宁德”建设、早日全面建成小康社会而努力奋斗！
